# The mediating role of work engagement between perceived organizational support and work competency among Chinese infection control practitioners

**DOI:** 10.3389/fpsyg.2026.1856236

**Published:** 2026-08-17

**Authors:** Wenjing Jiang, Li Cao, Chunli Song, Jun Li, Yuhan Zhang, Ping Zhou

**Affiliations:** 1 Department of Nursing, Sichuan Vocational College of Health and Rehabilitation, Zigong, China; 2 Department of Hospital Infection Management, Zigong First People's Hospital, Zigong, Sichuan, China; 3 Department of Operating Room, Zigong First People's Hospital, Zigong, China

**Keywords:** infection control practitioner, mediating effect, perceived organizational support, work competency, work engagement

## Abstract

**Background:**

Infection control practitioners (ICPs) play a fundamental role in preventing and controlling healthcare-associated infections. ICPs’ work competency is associated with work engagement and perceived organizational support (POS); however, the intercorrelations and mechanisms among Chinese ICPs remain unknown. Based on the job demands-resources (JD-R) model and conservation of resources (COR) theory, this study aimed to test the mediating effect of work engagement on the relationship between POS and work competency.

**Methods:**

A cross-sectional study was conducted among 340 ICPs from 87 hospitals in Sichuan Province, China, from June to September 2023. Data were collected via an online questionnaire that included basic characteristics, the perceived organizational support scale, the Utrecht Work Engagement Scale (UWES), and the Infection Control Competency Questionnaire for ICPs. Bootstrap methods, utilizing Hayes’s PROCESS Macro, were employed to test the mediation effect of work engagement on the relationship between POS and work competency.

**Results:**

The mean work competency, POS, and work engagement scores were 66.03 ± 11.11, 42.94 ± 8.09, and 73.89 ± 11.63, respectively. The mediation model demonstrated a robust association between POS and work competency, which was partially mediated by work engagement (total effect *c* = 0.739, 95% CI = 0.621–0.857; direct effect *c*’ = 0.475, 95% CI = 0.334–0.616; indirect effect ab = 0.264, 95% CI = 0.170–0.374).

**Conclusion:**

This study offers new insights into the relationship between POS and work competency, highlighting the mediating role of work engagement. Our findings suggest that enhancing ICPs’ POS and work engagement may be effective in improving their work competency, thereby enhancing patient safety and health outcomes.

## Background

Healthcare-associated infections (HAIs) represent a significant global health concern and are the second leading cause of death worldwide (Haque et al., 2020). The estimated prevalence of HAIs is 7% in developed countries and 10% in developing countries, contributing to high morbidity, mortality, and healthcare costs (Khan et al., 2017; Haque et al., 2018; World Health Organization, 2016). Infection control practitioners (ICPs), as the core forces in healthcare settings, play a fundamental role in the prevention and control of HAIs (Lipke et al., 2016). ICPs are involved in direct infection prevention and control activities as well as indirect surveillance, education, research, communication, and administration tasks (World Health Organization, 2020). To effectively protect the safety of medical staff and patients, ICPs must possess high levels of work competency to address the increasingly complex and demanding HAI challenges. ICPs’ work competency refers to “the knowledge, skills, and attitudes required to practice with an in-depth understanding of situations, use reasoning, critical thinking, reflection, and analysis to inform assessment and decision-making in the prevention and control of HAIs and antimicrobial resistance” (World Health Organization, 2020). ICPs’ work competency determines the implementation and quality of HAI management, and understanding the current state and determinants of work competency among ICPs is crucial for guiding subsequent improvement strategies and efforts.

A wide range of influencing factors have been identified in the literature that may affect ICPs’ work competency, including socio-demographic characteristics and work-related factors. Several studies indicate that certain socio-demographic factors are significantly associated with higher work competency among ICPs. Specifically, studies have shown that older age, female gender, and being married were associated with higher work competency (Zhao et al., 2024; Desta et al., 2018). Additionally, ICPs with longer work experience and higher education are more likely to hold senior professional titles and management positions with higher incomes, all of which contribute to enhanced competencies in infection prevention and control (Zhao et al., 2024; Desta et al., 2018). Furthermore, ICPs who work full-time in higher-level hospitals may have greater resources and responsibilities in managing HAIs, thereby exhibiting superior work competencies (Zhao et al., 2024; van Dijk et al., 2022).

Among work-related factors, perceived organizational support (POS) and work engagement are among the most widely reported contributors to work competency. POS refers to employees’ perceptions that their efforts and contributions are valued and their well-being is supported by their organizations (Eisenberger et al., 1986). POS is a crucial environmental resource for mitigating stress among individuals who struggle to adapt to their work environment, particularly when facing demanding tasks (Eisenberger et al., 2020). The association between POS and work competency has been well established, with a study among nurses in France showing that POS was positively associated with self-competency through affective commitment (Battistelli et al., 2016). Another study among ICPs in China also found that POS had a good predictive effect on work competency (Cui et al., 2023). Work engagement is technically defined as a positive, fulfilling, work-related state of mind characterized by vigor, dedication, and absorption (Schaufeli et al., 2002). Unlike transient emotions, it represents a pervasive affective-cognitive state. Vigor denotes high energy and resilience; dedication entails a sense of significance and challenge; and absorption is characterized by deep engagement in work tasks to the point that time passes rapidly. As a form of motivation, work engagement is vital in maximizing work potential and optimizing work effect (Motyka, 2018). Work engagement and work competency are frequently studied together as mutually reinforcing constructs in the workplace, with higher work engagement associated with higher work competency, and vice versa (Wei et al., 2018; Surya et al., 2023). Besides, accumulating evidence has consistently illustrated a positive association between POS and work engagement (Shan et al., 2023; Tian et al., 2023). Employees with higher work engagement demonstrate greater work competency by actively developing and improving work-related knowledge, skills, and attitudes (Hu et al., 2021).

Given extensive evidence of positive associations between POS and work competency, between work engagement and work competency, and between POS and work engagement across studies, we hypothesize that work engagement may mediate the relationship between POS and work competency. The mediating effect of work engagement in the relationship between POS and work competency may be well explained by various theoretical models. The job demands-resources (JD-R) model is one of the most dominant and heuristic models in work psychology that has been widely applied in research on occupational well-being (Demerouti et al., 2001). The JD-R model identifies two major job characteristics: job demands and job resources, which correspond to two distinct processes: the health impairment process and the motivational process, which are associated with two different health outcomes: decreased or increased well-being (Demerouti et al., 2001). Job demands are characterized by sustained physical and psychological input that consumes effort and energy, such as high workload and emotionally demanding communications, which initiate the health impairment process and bring about poor health outcomes (Schaufeli and Bakker, 2004). In contrast, job resources are intrinsic and extrinsic job characteristics that promote personal growth, learning, and development, helping individuals achieve work goals while offsetting the adverse effects of job demands (Bakker and Demerouti, 2007). Job resources initiate the motivational process, which is associated with improved work performance and better health outcomes. According to the JD-R model, POS is a beneficial job resource that enhances work engagement (the motivational process), which is in turn linked to better work-related performance, including work competency (Battistelli et al., 2016).

Beyond the JD-R model, the conservation of resources (COR) theory provides additional insight into the mediating role of work engagement in the relationship between POS and work competency (Hobfoll, 1988). The basic tenet of the COR theory is that resources tend to exist in clusters rather than in isolation, and individuals strive to obtain, create, foster, conserve, protect, and gain resources as much as possible to maximize resource gain and minimize resource loss (Hobfoll, 1988; Brotheridge and Lee, 2002; Gorgievski and Hobfoll, 2008). COR theory categorizes resources as internal (resources possessed by oneself or within one’s domain) and external resources (resources not possessed by oneself but external to it) (Hobfoll, 1988). External resources may help individuals enhance or develop their internal resources, as people are motivated to invest current resources to acquire additional resources (Hobfoll, 1988). According to the COR theory, POS is a powerful external resource that may contribute to the internal resource of work engagement, and the combined external and internal resources may achieve the maximal effect on improved work competency.

**Figure 1:**
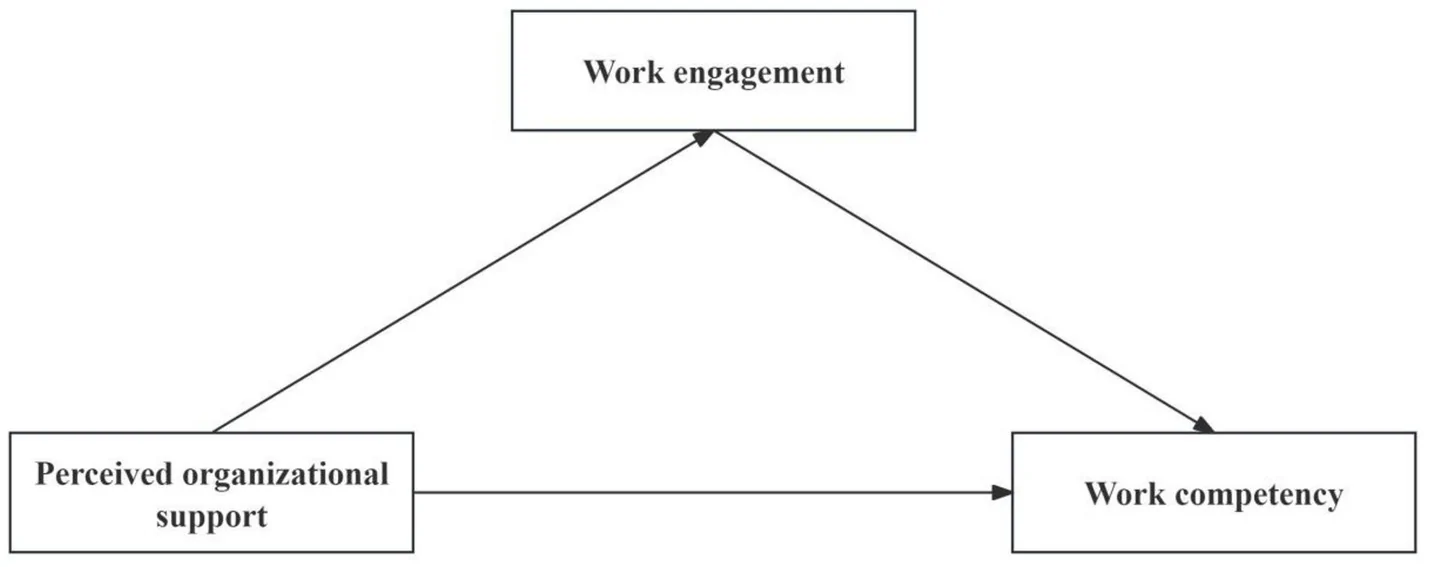
Hypothesized model of the mediating role of work engagement on the association between POS and work competency.

Although both empirical evidence and theoretical frameworks support the potential mediation model of POS, work engagement, and work competency, few studies have investigated these variables simultaneously. Some studies have examined mediation models using two of the three variables mentioned above, such as work engagement mediating the relationship between leadership and work competency (Wei et al., 2018) and psychological capital mediating the relationship between POS and work engagement (Tian et al., 2023). However, no study has explored the mediating effect of work engagement in the association between POS and work competency to date. In addition, most of the previous studies were conducted in Western countries, and much less is known about Asian countries such as China, which is highly burdened with HAIs. In China, two recent meta-analyses reported a pooled prevalence of HAIs of 3.13% (Wang et al., 2018) and estimated that HAIs increased medical expenditure by ¥24,881.37 (Liu et al., 2022). ICPs play a crucial role in healthcare in China, yet their work competency and the organizational contexts in which they practice are highly diverse and poorly documented. To the best of our knowledge, the relations among POS, work engagement, and competency among Chinese ICPs have not yet been confirmed. To address the research gap, we conducted the current study to investigate the work competency of Chinese ICPs and to examine the mediating role of work engagement in the relationship between POS and work competency. Based on the JD-R model and COR theory, we proposed the following hypotheses (Figure 1):

Hypothesis 1: POS was positively associated with work competency.

Hypothesis 2: Work engagement was positively associated with work competency.

Hypothesis 3: Work engagement mediated the association between POS and work competency.

In addition, previous literature reviews on the influencing factors of ICPs’ work competency have consistently shown that ICPs’ sociodemographic characteristics, such as age, gender, education, and years of work experience, are associated with their work competency (Brooks et al., 2021; Alhumaid et al., 2021; McCauley et al., 2021). Therefore, we included these variables in our investigation of the factors influencing ICPs’ work competency, employing multivariate regression analysis. We controlled for all significant confounding factors in the subsequent mediation analysis.

## Methods

### Setting and participants

A cross-sectional study was conducted among ICPs in Sichuan Province, China, from June to September 2023. A convenience sampling method was employed to recruit ICPs from 87 hospitals across 11 cities in Sichuan Province. The inclusion criteria of participants included: (1) age > 18, (2) registered health workers, (3) working in infection prevention and control management for at least 1 year, and (4) voluntary participation in this study. We excluded ICPs who were casual employees or off-duty due to sick leave, maternity leave, study abroad, or other reasons.

The sample size for this study was determined based on power analysis requirements for mediation modeling, following the recommendations of Fritz and MacKinnon (2007). Based on preliminary data and prior literature, the effect sizes for the paths in our mediation model were expected to be medium to large (Cohen’s *r* > 0.3). According to Fritz and MacKinnon’s (2007) power tables for the bias-corrected bootstrapping method, a sample size of at least 148 is required to reach a power of 0.80 for detecting medium-sized mediated effects. Our final sample of 340 participants provides more than sufficient power to detect the hypothesized effects and accounts for potential non-response or missing data.

### Data collection

The study was approved by the Institutional Review Board of Zigong First People’s Hospital (Approval No. 2023124). Data were collected through Sojump, one of China’s largest professional online survey platforms, which provides research services including questionnaire design, distribution, data collection, analysis, and result reporting (Zhang et al., 2017). Before the investigation, our research team sent study flyers via the online chatting platform WeChat to directors of infection prevention and control (IPC) centers in 11 cities. These directors then distributed the flyers to their respective work WeChat groups. Those interested in participating in this study can scan the QR code on WeChat to complete the survey. Participants were presented with an electronic version of informed consent explaining the study’s purpose, risks, and benefits via the smartphone or computer interface. The research was conducted anonymously, with all participants assigned a unique identification number for research purposes. Participants were informed that participation in the study was totally voluntary and they could withdraw at any time for any reason. They were also assured that their personal information would be kept confidential and that their responses would be accessible only to study personnel. All data collected from the survey and results from the analyses could only be used for research purposes and not for commercial purposes. After providing electronic informed consent, participants completed an online questionnaire that took approximately 10 min. To ensure data quality, the online survey was designed to require a response for every item, effectively eliminating missing values. After data collection, a manual screening process was conducted. Listwise deletion was applied to exclude invalid responses, such as those with logical inconsistencies or completion times of less than 300 s. A total of 348 questionnaires were submitted. After removing 8 invalid responses, 340 valid questionnaires were included in the analysis, yielding a valid response rate of 97.70% (340/348).

## Measurements

### Basic characteristics

Participants’ basic characteristics included gender, age, marital status, major, education level, professional title, years of working experience, monthly income (in CNY), position, whether they were full-time ICPs, and hospital level.

### POS

POS was measured using the brief Chinese version of the perceived organizational support scale, developed by Ling et al. (2006) and later modified by Meng (2016) to reflect the work situations of health workers in China. The scale comprises 12 items across three dimensions: support for work (5 items), concerns about interests (4 items), and value identity (3 items). Each item is scored on a 5-point Likert scale ranging from 1 (strongly disagree) to 5 (strongly agree). The total score ranges from 12 to 60, with a higher score indicating better perceived organizational support. In this study, the Cronbach’s *α* was 0.958.

### Work engagement

Work engagement was assessed using the Chinese version of the Utrecht Work Engagement Scale (UWES), initially developed by Schaufeli et al. (2002) and subsequently translated and adapted by Li et al. (2006). The scale comprises 16 items across three dimensions: work vigor (6 items), work dedication (5 items), and work absorption (5 items). Each item is scored on a 6-point Likert scale ranging from 1 (never) to 6 (always). The total score ranges from 16 to 96, with higher scores indicating greater work engagement. In this study, the Cronbach’s *α* was 0.933.

### Work competency

ICPs’ work competency was measured using the Infection Control Competency Questionnaire developed by Chen (2019) to assess the competency of infection prevention and control professionals in China. The questionnaire comprises 47 items across four major dimensions: basic skills (3 sub-dimensions, 12 items), organizational cooperation ability (3 sub-dimensions, 13 items), professional development ability (3 sub-dimensions, 13 items), and personal traits (2 sub-dimensions, 9 items). Each item was scored on a 7-point Likert scale ranging from 1 (strongly disapprove) to 7 (strongly approve). According to the original developer of the scale (Chen, 2019), the total score is calculated as follows, with weights assigned to each dimension: basic skills × 0.266 + organizational cooperation ability × 0.251 + professional development ability × 0.237 + personal traits × 0.245, with higher scores indicating better work competency. In this study, the Cronbach’s *α* was 0.988.

### Data analysis

All data analyses were conducted using IBM SPSS version 24.0. We first conducted Harman’s single-factor test to check for common method bias (Podsakoff et al., 2024). The results showed that 9 factors were generated, with the first factor accounting for 27.41% of the total variance, which was below the critical standard of 40–50% (Podsakoff et al., 2024). This preliminary test suggests that severe common method bias was not a major concern in the present data. However, we acknowledge that Harman’s single-factor test has methodological limitations and cannot definitively rule out common method variance. Frequencies and percentages were used to describe categorical variables, and means and standard deviations were used to describe the distributions of continuous variables.

For univariate analyses, we compared work competency across different demographic groups using *t*-tests or ANOVA. For demographic variables with ordinal characteristics (professional title, hospital level, monthly income, and working years), the Kruskal–Wallis *H* test was employed to compare differences in work competency, as it does not require the assumption of normality or homogeneity of variance. To control for the inflation of Type I error due to multiple testing across 11 demographic variables, a global Bonferroni correction was applied, resulting in an adjusted significance level of *α*’ = 0.0045 (0.05/11).

Pearson’s correlation coefficient (*r*) was used to explore the correlations between key variables, since they were normally distributed, as shown in the Q–Q plot (Supplementary file S1). Multiple linear regression analysis was used to investigate the independent factors associated with work competency, with all significant variables from the previous univariate analyses, after the global Bonferroni correction, included as independent variables. To ensure model stability, the assumptions of multiple linear regression were rigorously tested. Multi-collinearity was assessed using the variance inflation factor (VIF), with a value of less than 5.0 indicating the absence of multicollinearity (Akinwande et al., 2015). The independence of errors was evaluated using the Durbin–Watson test. Normality, linearity, and homoscedasticity were visually inspected using Normal P–P plots and scatter plots of standardized residuals. Cook’s distance was calculated to identify potential influential outliers. The practical significance of the regression models was assessed using the coefficient of determination (*R*^2^) for the overall model and semi-partial *R*^2^ (sr^2^) for individual variables. Semi-partial *R*^2^ represents the unique proportion of variance in the dependent variable explained by a specific independent variable after controlling for all other variables in the model.

Mediation analysis was performed using Hayes’ PROCESS macro (Model 4). This OLS-based path analysis approach was chosen due to the high internal consistency of the measurement instruments (Cronbach’s *α* > 0.90 for all constructs), which minimizes the bias associated with measurement error. The indirect effects were evaluated using 5,000 bootstrap samples to ensure robust standard errors and confidence intervals. Before conducting the mediation analysis, the assumptions of OLS regression were checked. Although the standardized residuals showed a departure from normality (Kolmogorov–Smirnov and Shapiro–Wilk tests, *p* < 0.001). Visual inspection of the histograms revealed that the distributions were approximately bell-shaped, despite the significant formal tests. In addition, Hayes’ PROCESS macro is robust to such violations because it uses nonparametric bootstrapping to estimate indirect effects. By generating 5,000 bootstrap samples, the analysis provides robust confidence intervals that do not rely on the assumption of normality. We examined the mediating role of work engagement in the relationship between POS and work competency, while controlling for all significant variables identified in the previous univariate analysis. The level of significance was set at 0.05 (two-sided). An effect was considered significant if its 95% bootstrap confidence interval from 5,000 bootstrap samples did not include zero.

## Results

### Work competency score by sample characteristics

Table 1 shows the sample characteristics and the distribution of work competency scores. A total of 340 participants completed the study, with a mean age of 38.44 ± 9.23 (range: 22–59 years). Most were female (92.1%), married (85.9%), with a nursing major (79.4%), holding an undergraduate degree (71.5%), in a technical position (70.9%), and working in tertiary hospitals (61.4%). A comparison of work competency scores by sample characteristics after applying the global Bonferroni correction (*α*’ = 0.0045) revealed significant differences in two variables: age (*p* = 0.003) and hospital level (*p* < 0.001). Other variables, including professional title and working years, did not reach the adjusted significance threshold and were therefore excluded from the subsequent multivariate analysis.

**Table 1:** Work competency score by sample characteristics (*N* = 340).

Variable	*N* (%)	Mean (S.D.)	*t/F* ^a^	*p*	Effect size
Gender			0.736	0.468	0.217^d^
Male	27 (7.9)	68.25 ± 11.69			
Female	313 (92.1)	65.84 ± 11.05			
Age (years)			4.822	0.003^b^	0.041^e^
≤30	63 (18.6)	61.63 ± 11.84			
>30~40	151 (44.4)	66.90 ± 11.02			
>40~50	82 (24.1)	66.22 ± 10.43			
>50	44 (12.9)	69.01 ± 10.07			
Marital status			2.846	0.059	0.017^e^
Married	292 (85.9)	66.03 ± 11.04			
Single	33 (9.7)	63.50 ± 12.02			
Divorced	15 (4.4)	71.71 ± 8.62			
Major			0.301	0.877	0.004^e^
Clinical medicine	44 (12.9)	65.32 ± 11.72			
Nursing	270 (79.4)	66.29 ± 11.03			
Public health	9 (2.7)	62.70 ± 9.59			
laboratory medicine	6 (1.8)	64.77 ± 9.47			
others	11 (3.2)	66.00 ± 13.59			
Education level			2.685	0.070	0.016^e^
Junior college or below	77 (22.6)	63.51 ± 11.29			
Undergraduate	243 (71.5)	66.91 ± 10.78			
Postgraduate or above	20 (5.9)	63.93 ± 11.83			
Professional title			3.415	0.016^c^	0.039^e^
Primary	99 (29.1)	64.14 ± 11.71			
Junior	153 (45.0)	65.68 ± 11.35			
Senior	88 (25.9)	68.79 ± 9.42			
Working years			2.890	0.016^c^	0.041^e^
0~5	43 (12.6)	63.04 ± 11.50			
>5~10	62 (18.2)	65.38 ± 11.24			
>10~15	91 (26.8)	64.54 ± 11.72			
>15~20	32 (9.4)	70.46 ± 10.42			
>20~25	37 (10.9)	65.35 ± 11.11			
>25	75 (22.1)	68.55 ± 9.48			
Monthly income (CNY)			2.741	0.045^c^	0.032^e^
≤3,000	16 (4.7)	61.3 ± 11.69			
>3,000~5,000	120 (35.3)	64.54 ± 11.83			
>5,000~7,000	114 (33.5)	66.45 ± 10.63			
>7,000~10,000	68 (20.0)	69.19 ± 9.77			
>10,000	22 (6.4)	65.71 ± 11.20			
Position			−0.609	0.543	−0.073^d^
Management position	99 (29.1)	65.46 ± 10.73			
Technical position	241 (70.9)	66.27 ± 11.27			
Full-time ICP			0.060	0.952	0.007^d^
Yes	202 (59.4)	66.06 ± 10.72			
No	138 (40.7)	65.99 ± 11.68			
Hospital level			17.146	<0.001^b^,^c^	0.092^e^
Primary	53 (15.6)	58.19 ± 10.54			
Secondary	78 (23.0)	67.58 ± 10.06			
Tertiary	209 (61.4)	67.44 ± 10.82			

a *t* refers to a *t*-statistic used to compare means between two groups, which is the ratio of the difference between sample means to the standard error of the difference; *F* refers to the *F*-statistic, which is the ratio of the variances between groups to the variance within groups (in ANOVA).

b Indicates statistical significance using a Bonferroni-adjusted alpha of 0.0045.

c Kruskal–Wallis tests were performed for ordinal variables, yielding consistent results with ANOVA.

d Cohen’s *d*.

e Partial *η*^2^.

### Correlations among key variables

The mean work competency, POS, and work engagement scores were 66.03 ± 11.11, 42.94 ± 8.09, and 73.89 ± 11.63, respectively. Correlation analysis showed that POS was positively correlated with work engagement (*r* = 0.574, *p* < 0.01) and work competency (*r* = 0.526, *p* < 0.01). Work engagement was also positively correlated with work competency (*r* = 0.547, *p* < 0.01) (Table 2).

**Table 2:** Mean scores and correlations among the study variables (*N* = 340).

Variable	1	2	3
1. Work competency	1.000		
2. POS	0.526^***^	1.000	
3. Work engagement	0.547^***^	0.574^***^	1.000
Mean (S.D.)	66.03 ± 11.11	42.94 ± 8.09	73.89 ± 11.63

^***^p < 0.001. POS, perceived organizational support.

### Factors associated with work competency

A multiple linear regression analysis was computed with work competency as the dependent variables and all significant variables in the univariate analysis as independent variables. Regression diagnostics confirmed that the model met all necessary assumptions (Supplementary file S2). The VIF values for all variables ranged from 1.008 to 1.611, confirming the absence of multicollinearity. The Durbin-Watson value of 1.934 confirmed the independence of residuals. Visual inspection of the P–P plot and residual scatter plots confirmed normality and homoscedasticity. Furthermore, the maximum Cook’s distance (0.086) was well within the acceptable range (all cases <1.0), suggesting that the model was not significantly biased by influential outliers.

As shown in Table 3, the final model significantly explained work competency (Adjusted *R*^2^ = 0.432, *p* < 0.001). The results identified four significant factors that were associated with higher work competency: older age (*B* = 1.713, *p* = 0.001), higher hospital level (*B* = 3.126, *p* < 0.001), higher POS (*B* = 0.475, *p* < 0.001), and higher work engagement (*B* = 0.305, *p* < 0.001) (Table 3). The sr^2^ values indicate that POS and work engagement uniquely accounted for 7.5 and 6.4% of the variance, respectively.

**Table 3:** The factors associated with work competency by the multiple linear regression model (*N* = 340).

Variable	VIF	B	S.E.	*β*	*t*	*p*	95% CI	sr^2^
Constant		11.452	3.485		3.286	0.001	(4.597, 18.306)	
Age	1.099	1.713	0.521	0.142	3.291	0.001	(0.689, 2.737)	0.018
Hospital level	1.008	3.126	0.612	0.211	5.105	<0.001	(1.922, 4.331)	0.044
POS	1.604	0.475	0.072	0.346	6.633	<0.001	(0.334, 0.616)	0.075
Work engagement	1.611	0.305	0.05	0.319	6.115	<0.001	(0.207, 0.403)	0.064

Adjusted *R*^2^ = 0.432. VIF, variance inflation factor. sr^2^, semi-partial *R*^2^.

### Mediation analysis

**Figure 2:**
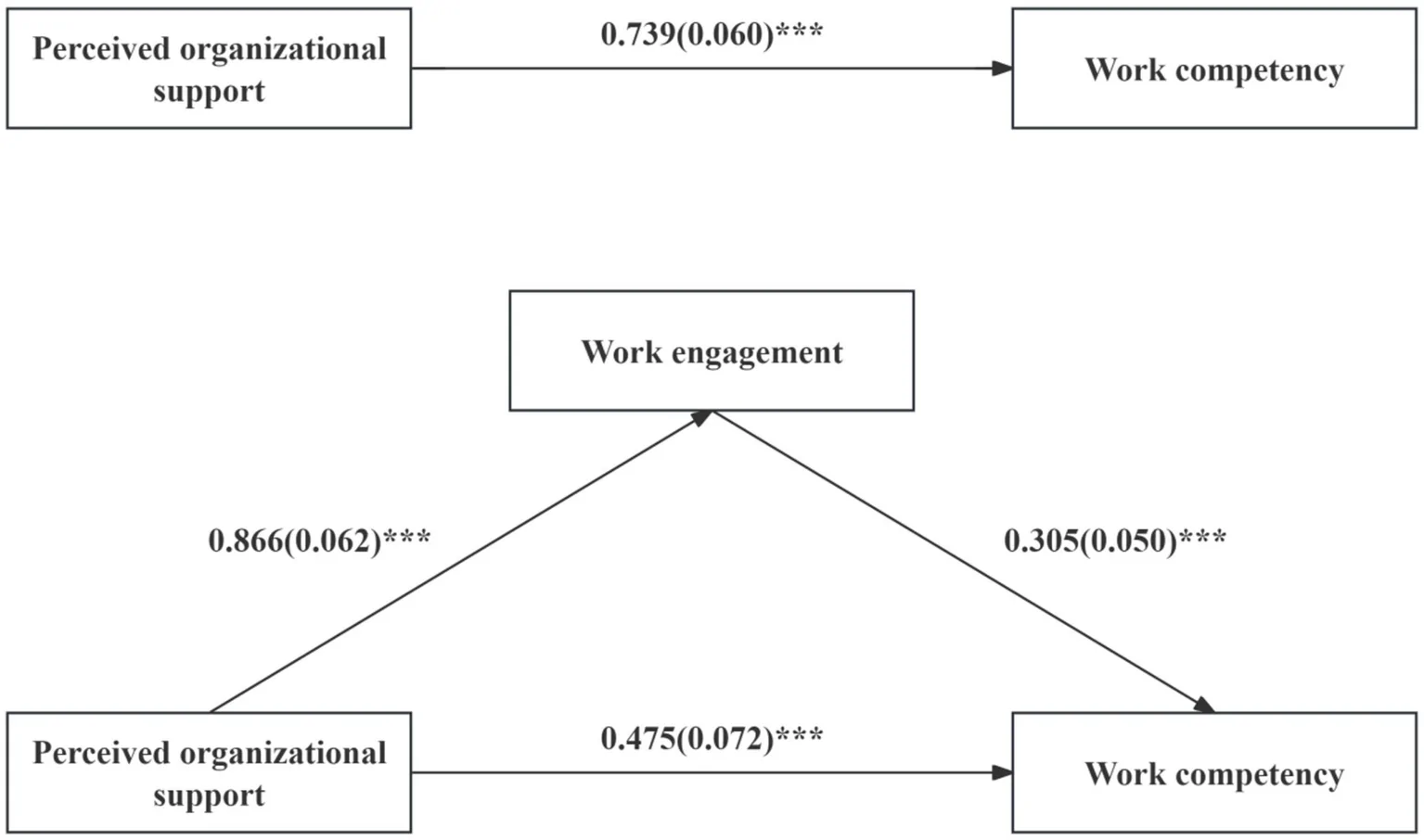
Mediation model of the effect of work engagement on the relationship between POS and competency (*N* = 340), ^***^p < 0.001.

Table 4, Figure 2 present the results of the mediation analysis, which examines the mediating effect of work engagement on the association between POS and work competency. The results indicated that work engagement significantly mediated the relationship between POS and work competency. The standardized total effect of POS on work competency was 0.538 (*p* < 0.001), supporting Hypothesis 1. After accounting for the mediator, the standardized direct effect remained significant (*β* = 0.346, *p* < 0.001). The standardized indirect effect through work engagement was 0.192 (95% CI: 0.125–0.266), indicating partial mediation. These results supported Hypotheses 2 and 3. To assess the magnitude of this mediation, the proportion mediated was calculated, showing that work engagement accounted for 35.7% of the total effect. This suggests that while POS has a strong direct link to competency, a substantial portion of this association is realized through work engagement. Comparing the standardized coefficients reveals that the direct effect of POS on competency is stronger than the indirect effect mediated by work engagement.

**Table 4:** Mediation analysis results (*N* = 340).

Effect	B	S.E.	*β*	*t*	*p*	95% CI
Indirect effect (*a* × *b*)	0.264	0.053	0.192	–	–	(0.170, 0.374)
Direct effect (*c*’)	0.475	0.071	0.346	6.634	<0.001	(0.334, 0.616)
Total effect (*c*)	0.739	0.060	0.538	12.298	<0.001	(0.621, 0.857)

*B* represents the unstandardized coefficient. *β* represents the standardized coefficient. Confidence intervals for the indirect effect were calculated using bootstrapping (5,000 samples).

## Discussion

This is the first study to investigate the status of work competency and to explore the relationship among POS, work engagement, and work competency among Chinese ICPs, using a mediation model. Our results showed that ICPs had an average work-competency score of 66.03 ± 11.11. Consistent with the JD-R model and COR theory, POS was positively associated with work competency, and this association was partially mediated by work engagement. Our findings offer fresh insights into ICPs’ work competency and provide essential implications for guiding future intervention programs to enhance their work competency by promoting POS and work engagement.

In our study, the total score for ICPs’ work competency was 66.03 ± 11.11 out of 160, indicating a moderate to high level of competency. The work competency score in our study was higher than that of previous studies using the same instrument among ICPs in other parts of China, such as Jilin Province (Chen, 2019), Anhui Province (Xu et al., 2022), and Sichuan Province (Huang et al., 2023). One explanation may be that our study was conducted more recently, when ICPs’ work competency significantly improved over time due to increased attention to and input on HAI control and ICP strengthening in China. Additionally, in response to the global COVID-19 pandemic, the Chinese government has strengthened the education and training of ICPs in healthcare settings at all levels nationwide to effectively prevent and control the pandemic (Huang L. et al., 2020; Huang X. et al., 2020), thereby enhancing their work competency.

Further multivariate regression analysis revealed that hospital level and age were significantly and positively associated with ICPs’ work competency, consistent with previous studies (Brooks et al., 2021; Alhumaid et al., 2021; McCauley et al., 2021). Compared with lower-level hospitals, higher-level hospitals generally have stronger leadership support, more comprehensive policies and regulations, and better resources for HAI prevention and control, which are associated with higher work competency among IPCs (Brooks et al., 2021; Alhumaid et al., 2021; McCauley et al., 2021). In addition, ICPs with older age gain more knowledge and skills in HAI prevention and control through prior work experience, which enables them to respond more effectively to workplace challenges and enhances their work competency (Brooks et al., 2021; Alhumaid et al., 2021; McCauley et al., 2021). Notably, the ICPs’ work competency scores should be interpreted with caution, as our sample was predominantly female, who may have different work competencies than males. However, the gender composition of ICPs in our study was consistent with previous studies in China since nurses are the major workforce involved in HAI prevention and control in China (Tian et al., 2023). In addition, our analysis revealed no significant difference in work competency scores by gender, consistent with a recent literature review indicating that gender is not associated with compliance with infection control measures (Brooks et al., 2021).

As hypothesized, a significant positive relationship was found between POS and work competency; specifically, ICPs with higher POS demonstrated better work competency, consistent with similar studies (Battistelli et al., 2016; Cui et al., 2023). ICPs who receive more support from their organization would feel valued, affirmed, and accepted. Thus, they would work hard and contribute their strength to achieve organizational goals (Eisenberger et al., 1986). In addition, our study showed that ICPs’ work engagement partially mediated the relation between POS and work competency; that is, higher POS was associated with higher work engagement, which, in turn, was associated with better work competency. This finding was also consistent with previous studies, which have shown that strong organizational support is correlated with stronger work motivation and engagement, which are in turn associated with effective job performance and high work competency (Battistelli et al., 2016).

Consistent with the JD-R model (Demerouti et al., 2001), our study identifies POS as a beneficial extrinsic job resource that may facilitate the achievement of work goals and support personal goals. Specifically, POS was associated with improved work engagement through the motivational process, which, in turn, is related to positive work-related outcomes, such as enhanced work competency. Since this study did not include job demand variables, we were unable to explore whether POS was associated with improved work competency through its protective roles in buffering against the adverse effects of job demand on work competency, which warrants further study. In addition, our mediation model provides strong support for the COR theory by demonstrating that individuals with higher POS levels also exhibit higher work engagement, which, in turn, is associated with improved work competency. Consistent with the COR theory, POS can be viewed as an external resource, and individuals with higher POS levels may thereby cultivate additional internal resources, which are associated with improved work engagement. Finally, individuals having both external resources (e.g., POS) and internal resources (e.g., work engagement) may be beneficial for achieving these resources to achieve their work goals, which is also associated with improved work competency.

Our findings have significant implications for guiding future intervention programs aimed at enhancing ICPs’ work competency. Strengthening POS could be a key strategy to achieve this goal. Specifically, multiple actionable strategies are recommended to help healthcare organizations improve POS to enhance work engagement and competency among ICPs. First and foremost, leadership and management support are pivotal to the development and implementation of policies and regulations aimed at improving ICPs’ work engagement and competency. Additionally, ongoing training curricula and educational programs should be provided to ICPs to ensure sustained personal development and career advancement. Education and training not only help improve ICPs’ knowledge and skills in HAI prevention and control but also may promote confidence and self-efficacy in performing their work, which are associated with improved work competency. Other strategies to improve POS include raising salaries and strengthening welfare for ICPs, increasing the workforce engaged in HAI prevention and control, and reducing ICP workload, all of which could contribute to improving work engagement and work competency among ICPs. In addition, for ICPs with inadequate organizational support, enhancing their work engagement may also be effective in improving their work competency.

## Limitations

The study has several limitations that warrant mention. First, the cross-sectional nature of this study precludes inferring causal relationships. While our mediation model (POS → work engagement → work competency) is grounded in Social Exchange Theory and the JD-R model, the possibility of reverse causality cannot be ruled out. For instance, ICPs with higher competency may receive more recognition, thereby reporting higher POS. Future longitudinal or experimental studies are required to confirm the directional pathways identified in this study. Second, the data were collected in Sichuan Province, China, which may limit the generalizability of the findings to other areas. Future studies could expand the scope of data collection. Third, this study employed a convenience sampling design, which limits the generalizability of the findings. Specifically, recruitment through IPC directors may have introduced institutional gatekeeping bias, and the voluntary nature of participation led to self-selection bias. Together, these likely resulted in a sample with systematically higher levels of POS, engagement, and competency, potentially inflating the observed associations. Future research should use stratified random sampling across diverse regions to validate these findings. Fourth, our study sample was predominantly female, who may differ from males in their work experiences. Future studies may consider validating our findings with a more gender-balanced sample. Fifth, due to the observational nature of the study, there is a possibility of residual or unmeasured confounding, such as organizational culture and workload. Future studies should collect data on additional relevant variables to mitigate such confounding. Sixth, the reliance on self-reported measures in a cross-sectional survey may increase the risk of common method bias. Although preliminary analysis using Harman’s single-factor test did not indicate severe common method variance, this test has known limitations. Therefore, the potential for common method bias cannot be completely ruled out. Future studies should use multiple data sources or a time-lagged design to better mitigate this risk. Seventh, the Cronbach’s alpha coefficients in this study were exceptionally high (e.g., 0.988 for work competency), indicating excellent internal consistency. However, values approaching 1.0 may also suggest item redundancy, which could inflate reliability estimates. Although validated scales were used, this potential redundancy warrants consideration when interpreting the strength of the observed associations. Eighth, although we confirmed the overall robustness of the mediation model by controlling for socio-demographic variables, future studies may consider conducting subgroup analyses to gain a deeper understanding of the model’s stability across different demographic groups. Finally, although the scales used in this study exhibited high reliability, the mediation model was analyzed using observed variables rather than latent constructs. While OLS-based mediation analysis (PROCESS) is a validated and widely accepted approach, it does not explicitly partition measurement error as in structural equation modeling (SEM). Future studies with larger samples could consider using SEM to further verify these associations.

## Conclusion

Our study revealed that ICPs’ work competency was moderate to high, with room for improvement in specific dimensions. POS was positively correlated with work competency both directly and indirectly through the mediation role of work engagement. Our study provides new insights into the relationship between POS and work competency, elucidating the mediating role of work engagement. Our findings have important implications for improving the work competency of ICPs in healthcare settings. Healthcare organizations should take multiple measures to support ICPs’ self-identity, self-worth, and POS. Moreover, promoting ICPs’ work engagement for those with inadequate POS may also be associated with improved work competency.

## Data Availability

The raw data supporting the conclusions of this article will be made available by the authors, without undue reservation.
