# Supplementary material for: The mediating role of work engagement between perceived organizational support and work competency among Chinese infection control practitioners
Source: Front Psychol. 2026 Aug 17;17:1856236. doi: 10.3389/fpsyg.2026.1856236 (PMC13526612; doi:10.3389/fpsyg.2026.1856236)
Supplement: Supplementary file 1 [file Supplementary_file_2.docx]

**Supplementary File S2: Multiple Linear Regression Analysis and Diagnostics**

This file contains the complete statistical output for the final multiple linear regression model predicting work competency, including model summary, ANOVA, coefficients, collinearity diagnostics, residual statistics, and diagnostic plots. This output corresponds to Table 3 and the associated regression diagnostics mentioned in the main manuscript.

**1. Regression Model Specification**

| Entered**/**Removed Variables**^a^** | | | |
| --- | --- | --- | --- |
| Model | Variables Entered | Variables Removed | Method |
| 1 | POS, Hospital level, Age, Work engagement^b^ | . | Enter |
| a. Dependent Variable: work competency | | | |
| b. All requested variables entered. | | | |

**2. Model Summary**

| **模型摘要^b^** | | | | |
| --- | --- | --- | --- | --- |
| Model | R | R Square | Adjusted R Square | Std. Error of the Estimate |
| 1 | .658^a^ | .432 | .426 | 8.416259 |
| a. Predictors: (Constant), POS, Hospital level, Age, Work engagement | | | | |
| b. Dependent Variable: work competency | | | | |

**3. Analysis of Variance (ANOVA)**

| **ANOVA^a^** | | | | | | |
| --- | --- | --- | --- | --- | --- | --- |
| Model | | Sum of Squares | df | Mean Square | F | Sig. |
| 1 | Regression | 18080.511 | 4 | 4520.128 | 63.813 | .000^b^ |
|  | Residual | 23729.195 | 335 | 70.833 |  |  |
|  | Total | 41809.706 | 339 |  |  |  |
| a. Dependent Variable: work competency | | | | | | |
| b. Predictors: (Constant), POS, Hospital level, Age, Work engagement | | | | | | |

**4. Regression Coefficients and Significance**

| **Coefficients^a^** | | | | | | | | | | |
| --- | --- | --- | --- | --- | --- | --- | --- | --- | --- | --- |
| Model | | Unstandardized Coefficients | | Standardized Coefficients Beta | t | Sig. | 95.0% Confidence Interval for B | | Collinearity Diagnostics | |
|  |  | B | Std. Error | Beta |  |  | Lower Bound | Upper Bound | Tolerance | VIF |
| 1 | (Constant) | 11.452 | 3.485 |  | 3.286 | .001 | 4.597 | 18.306 |  |  |
|  | Age | 1.713 | .521 | .142 | 3.291 | .001 | .689 | 2.737 | .910 | 1.099 |
|  | Hospital level | 3.126 | .612 | .211 | 5.105 | .000 | 1.922 | 4.331 | .992 | 1.008 |
|  | Work engagement | .305 | .050 | .319 | 6.115 | .000 | .207 | .403 | .621 | 1.611 |
|  | POS | .475 | .072 | .346 | 6.633 | .000 | .334 | .616 | .624 | 1.604 |
| a. Dependent Variable: work competency | | | | | | | | | | |

**5. Collinearity Diagnostics (Variance Proportions)**

| **Collinearity Diagnostics^a^** | | | | | | | | |
| --- | --- | --- | --- | --- | --- | --- | --- | --- |
| Model | Dimension | Eigenvalue | Condition Index | Variance Proportions | | | | |
|  |  |  |  | (Constant) | Age | Hospital level | Work engagement | POS |
| 1 | 1 | 4.786 | 1.000 | .00 | .00 | .00 | .00 | .00 |
|  | 2 | .120 | 6.317 | .00 | .81 | .07 | .00 | .02 |
|  | 3 | .070 | 8.259 | .01 | .01 | .83 | .02 | .05 |
|  | 4 | .014 | 18.428 | .72 | .13 | .07 | .00 | .57 |
|  | 5 | .010 | 21.685 | .27 | .04 | .03 | .97 | .37 |
| a. Dependent Variable: work competency | | | | | | | | |

**6. Residuals Statistics**

| **Residuals Statistics^a^** | | | | | |
| --- | --- | --- | --- | --- | --- |
| Statistic | Minimum | Maximum | Mean | Std. Deviation | N |
| Predicted Value | 41.52673 | 81.88243 | 66.03305 | 7.303071 | 340 |
| Std. Predicted Value | -3.356 | 2.170 | .000 | 1.000 | 340 |
| Standard Error of Predicted Value | .554 | 2.972 | .978 | .292 | 340 |
| Adjusted Predicted Value | 41.42559 | 81.93681 | 66.03779 | 7.310063 | 340 |
| Residual | -33.356701 | 23.182587 | .000000 | 8.366458 | 340 |
| Std. Residual | -3.963 | 2.755 | .000 | .994 | 340 |
| Studentized Residual | -4.016 | 2.763 | .000 | 1.001 | 340 |
| Deleted Residual | -34.243534 | 23.321665 | -.004736 | 8.491851 | 340 |
| Studentized Deleted Residual | -4.110 | 2.791 | -.001 | 1.005 | 340 |
| Mahalanobis Distance | .472 | 41.272 | 3.988 | 3.502 | 340 |
| Cook's Distance | .000 | .086 | .003 | .007 | 340 |
| Centered Leverage Value | .001 | .122 | .012 | .010 | 340 |
| a. Dependent Variable: work competency | | | | | |

**7. Regression Diagnostic Plots**

**7.1 Histogram of Standardized Residuals**

Note. Dependent Variable: work competency

**7.2 Normal P-P Plot of Standardized Residuals**

Note. Dependent Variable: work competency

**7.3 Scatterplot of Standardized Residuals vs. Standardized Predicted Values**

Note. Dependent Variable: work competency
