# Supplementary material for: The mediating role of work engagement between perceived organizational support and work competency among Chinese infection control practitioners
Source: Front Psychol. 2026 Aug 17;17:1856236. doi: 10.3389/fpsyg.2026.1856236 (PMC13526612; doi:10.3389/fpsyg.2026.1856236)
Supplement: Supplementary file 2 [file Supplementary_file_1.doc]

Figure 1. Q-Q plot for work engagement

Figure 2. Q-Q plot for POS

Figure 3. Q-Q plot for work competency
